# Supplementary material for: Advantages of eutectic alloys for creating catalysts in the realm of nanotechnology-enabled metallurgy
Source: Nat Commun. 2019 Oct 11;10:4645. doi: 10.1038/s41467-019-12615-6 (PMC6789138; doi:10.1038/s41467-019-12615-6)
Supplement: Supplementary file 1 — Supplementary Information [file 41467_2019_12615_MOESM1_ESM.pdf]

Supplementary Information:

Advantages of eutectic alloys for creating catalysts in the realm of nanotechnology-enabled metallurgy

Jianbo Tang[1], Rahman Daiyan[1], Mohammad B. Ghasemian[1], Shuhada A. Idrus-Saidi[1], Ali Zavabeti[2,3], Torben Daeneke[2], Jiong Yang[1], Pramod Koshy[4], Soshan Cheong[5], Richard D. Tilley[5,6,7], Richard B. Kaner[8,9], Rose Amal[1] & Kourosh Kalantar-Zadeh[1*]

[1] School of Chemical Engineering, University of New South Wales (UNSW), Sydney, NSW 2052, Australia

[2] School of Engineering, RMIT University, Melbourne, VIC 3001, Australia

[3] College of Material Science and Technology, Nanjing University of Aeronautics and Astronautics, 29 Jiangjun Ave, 211100 Nanjing, China

[4] School of Materials Science and Engineering, UNSW, Sydney, NSW 2052, Australia

[5] Mark Wainwright Analytical Centre, UNSW, Sydney, NSW 2052, Australia

[6] School of Chemistry, UNSW, Sydney, NSW 2052, Australia

[7] Australian Centre for NanoMedicine, UNSW, Sydney, NSW 2052, Australia

[8] Department of Chemistry and Biochemistry, University of California, Los Angeles (UCLA), Los Angeles, CA 90095, USA

[9] Department of Materials Science and Engineering, UCLA, Los Angeles, CA 90095, USA

*Correspondence to K.K.-Z. (email: k.kalantar-zadeh@unsw.edu.au)

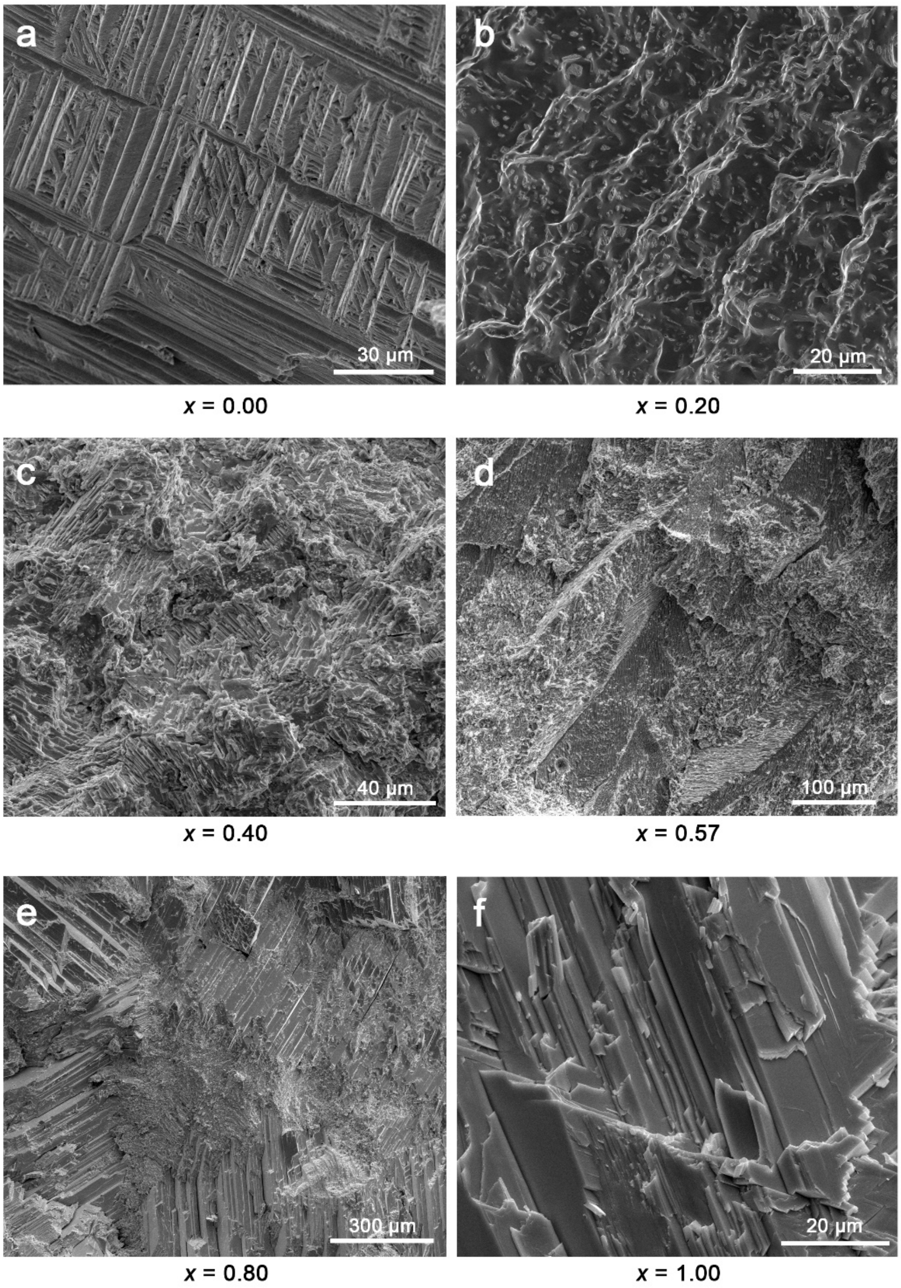

Supplementary Figure 1. Additional SEM images of the microstructures of the Bi_xSn_{1-x} bulk alloy samples and the Sn, Bi metals.

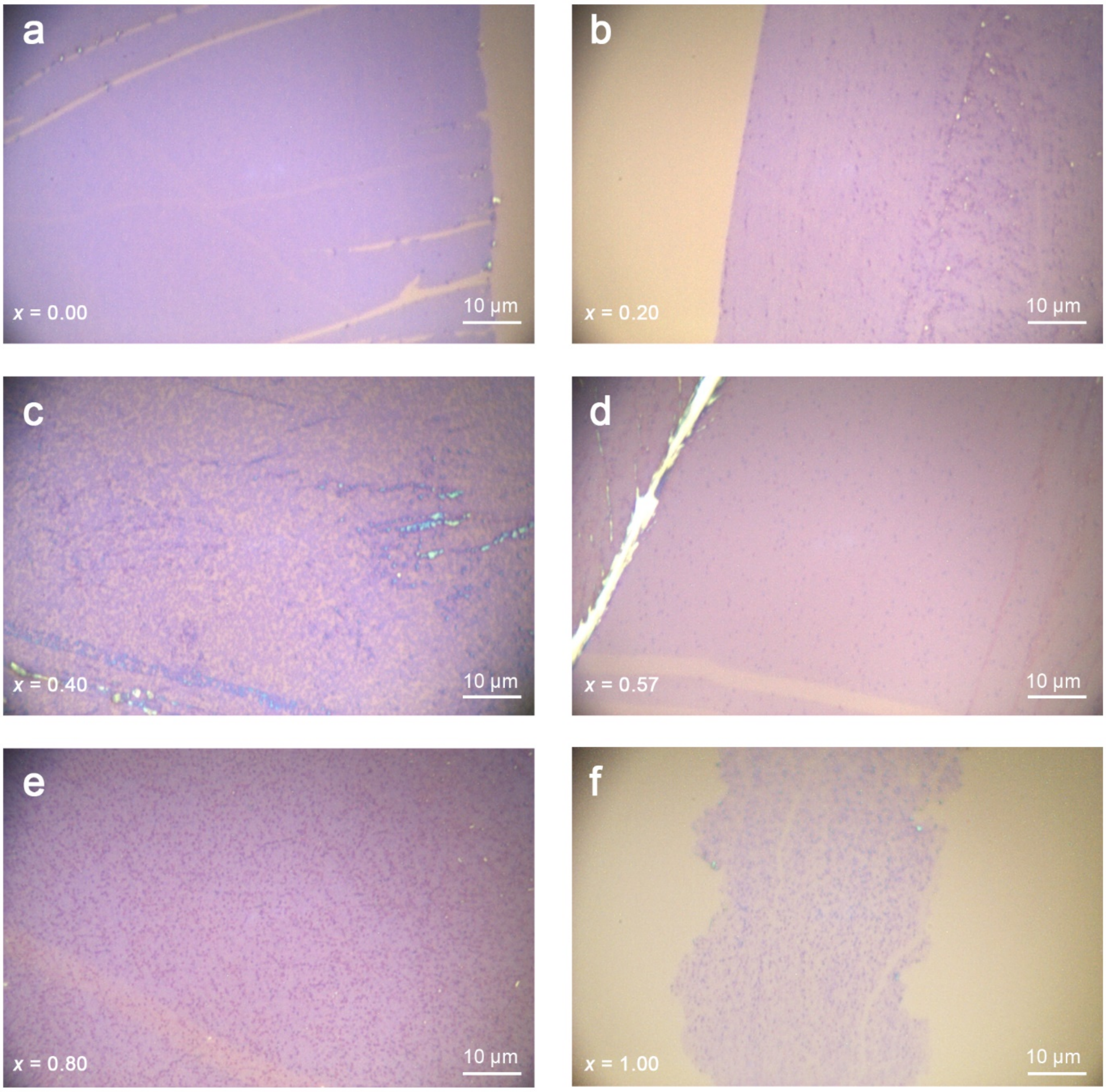

Supplementary Figure 2. Optical images of surface oxide layers for Raman tests. The oxide layers are touch-printed from liquid Bi_xSn_{1-x} bulk alloys in ambient air using silicon substrates.

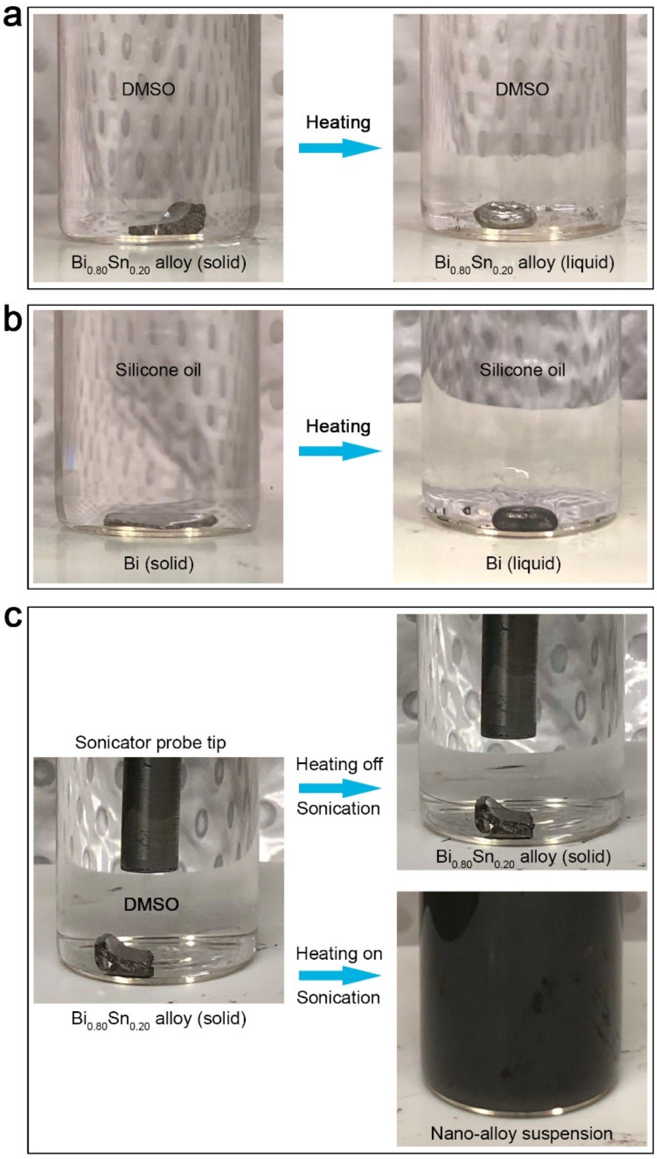

Supplementary Figure 3. Demonstration of the liquefied state of the samples and the necessarily of heating. **a** The $Bi_{0.80}Sn_{0.20}$ sample in DMSO solvent. **b** The Bi metal control sample in silicone oil. **c** Comparison of sonicating with and without heating to show that heating is necessary to fragment the liquid alloy into droplets. The hotplate is set to 400°C and its surface temperature is measured to be 397.4 ± 0.4 °C by an infrared thermometer.

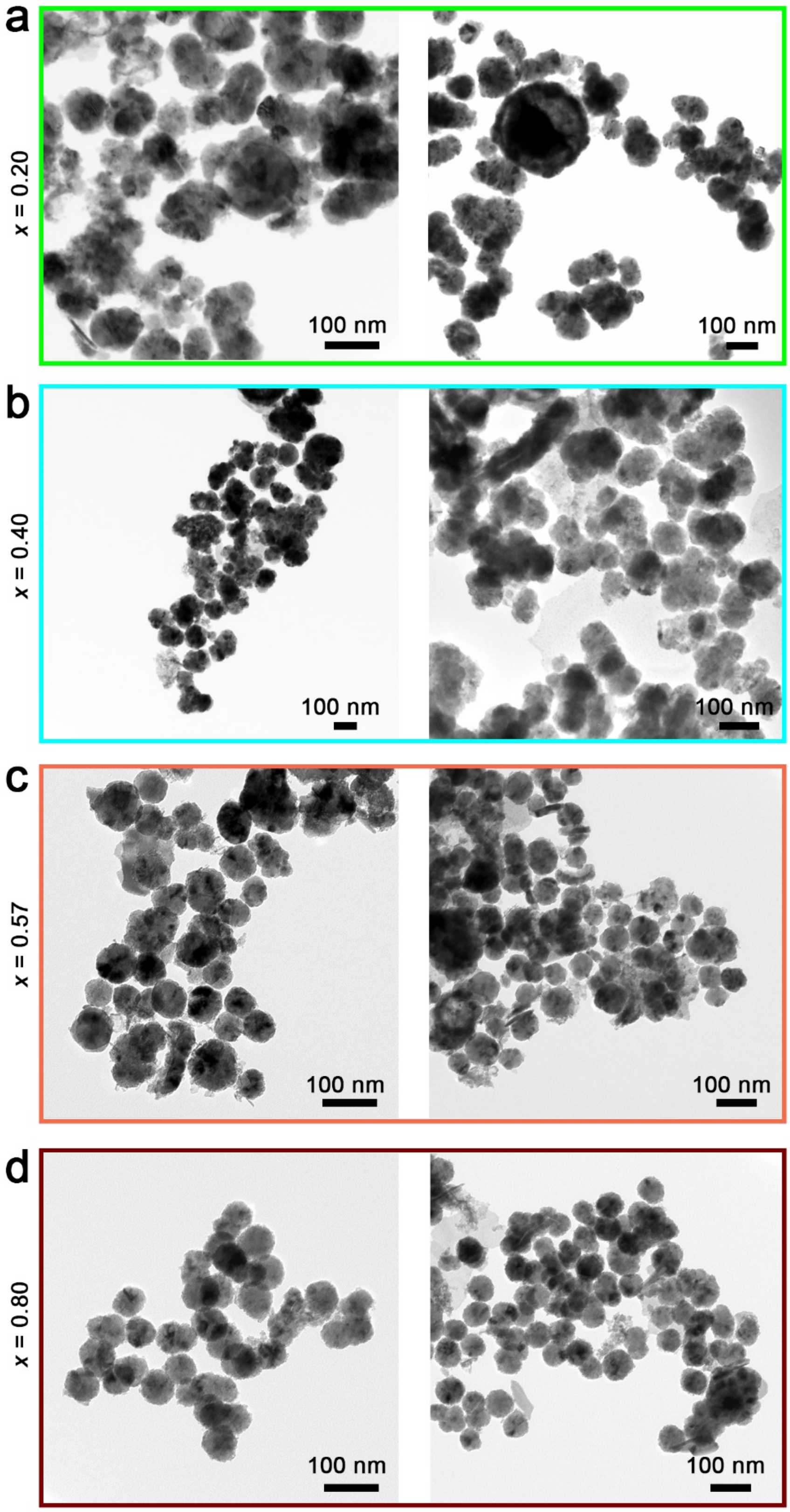

Supplementary Figure 4. Additional TEM images of the Bi_xSn_{1-x} nano-alloy samples prepared in this study. Some large particles typically > 200 nm are occasionally seen.

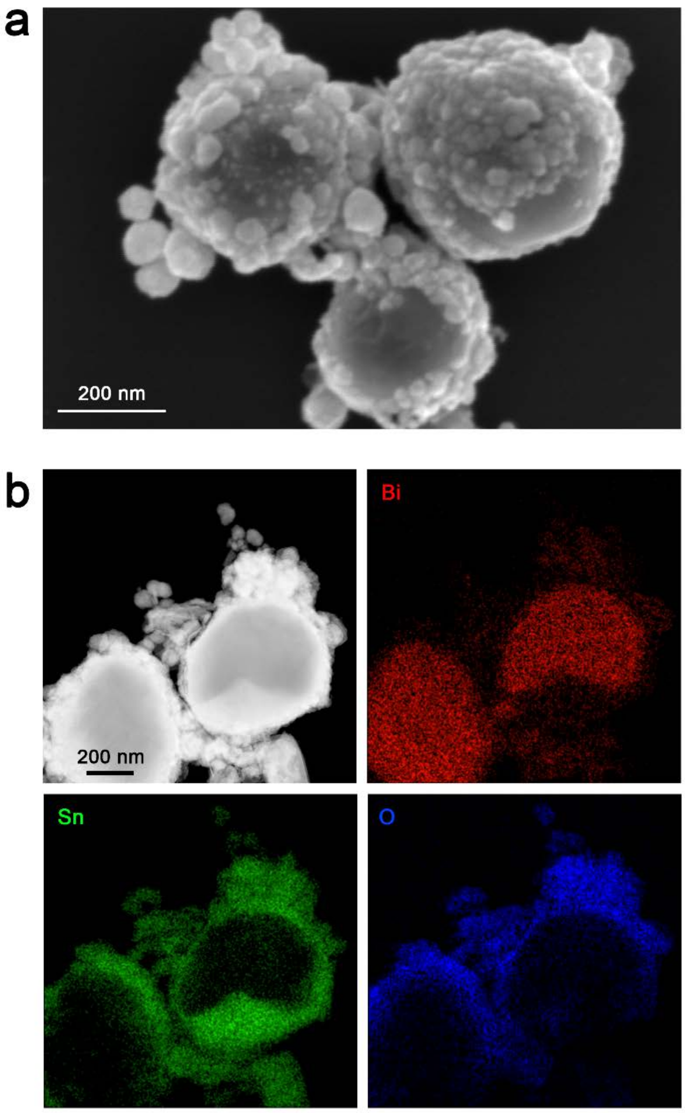

Supplementary Figure 5. a SEM images of the large particles that are usually partially coated by small particles to form core-shell structures. **b** TEM image and STEM-EDX mapping showing the element distribution in the Bi-rich cores and the shells composed of small particles.

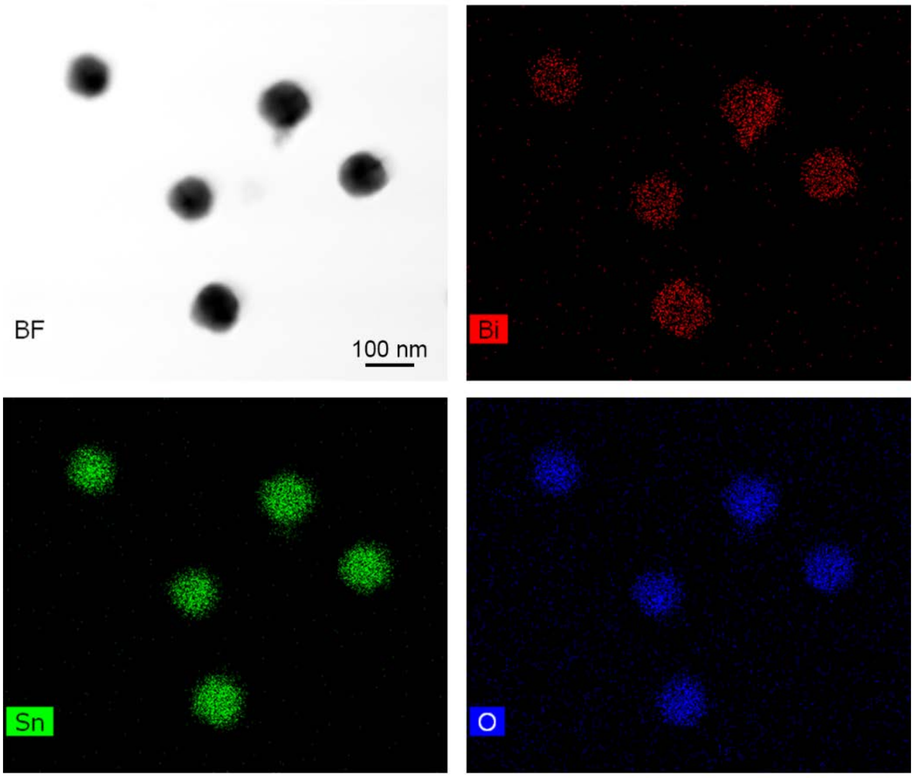

Supplementary Figure 6. Additional TEM-EDX mapping results of small particles of the eutectic nano-alloy sample.

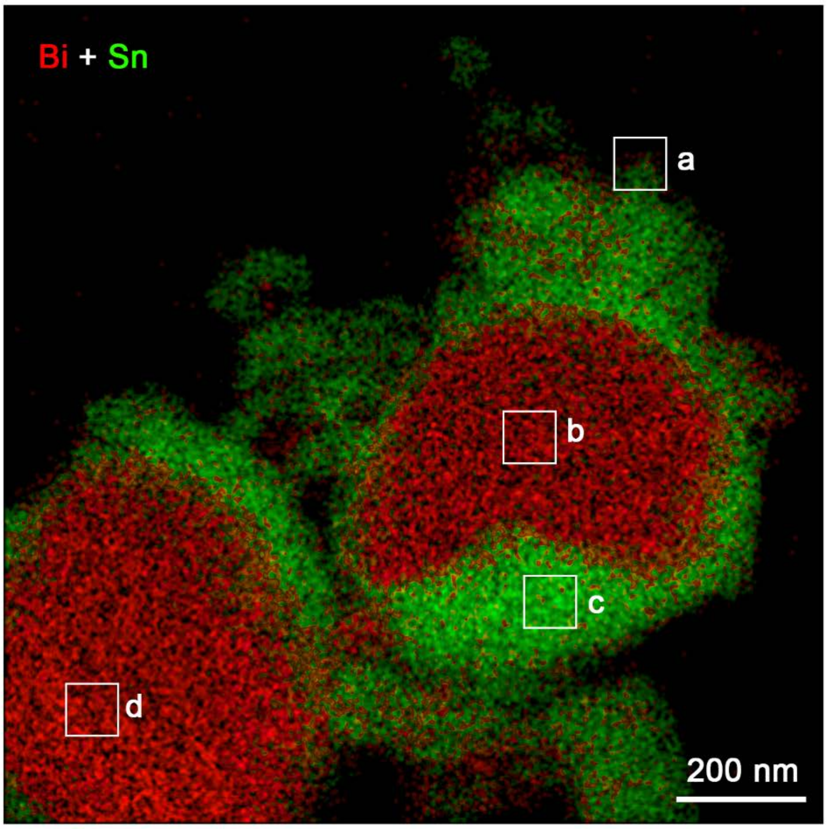

Element	Atom %			
	**a**	**b**	**c**	**d**
Sn	91	30	95	10
Bi	9	70	5	90

Supplementary Figure 7. Relative Bi-Sn atomic ratio in the small particle (a), Bi-rich cores of the large particles (b, d), and the small-particle-containing shell (c). The values are obtained from EDX spectra.

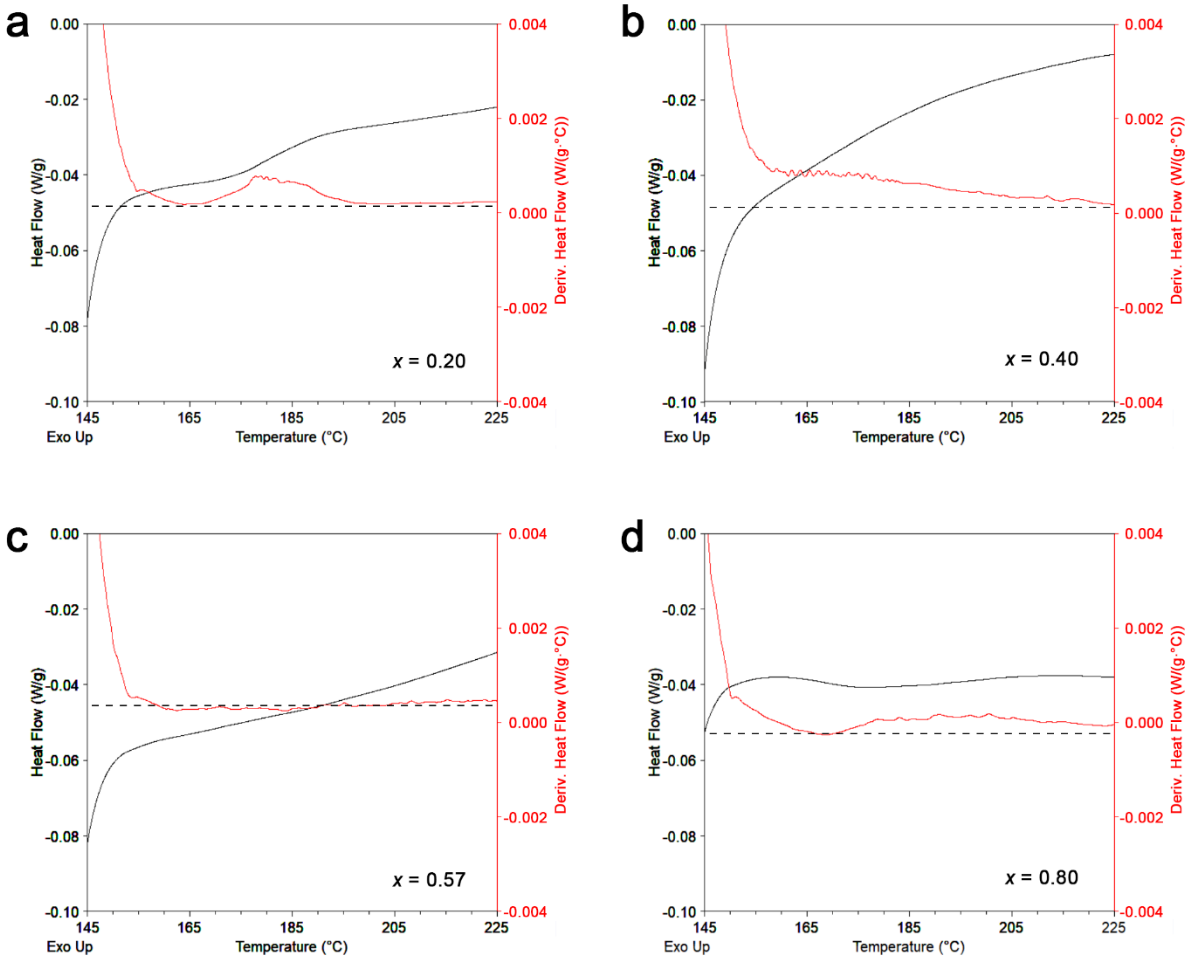

Supplementary Figure 8. Plots of heat flow (black curve) and its temperature derivative (red curve) for the different Bi-Sn nano-alloy samples shown in the insets of Fig. 3k in the main text. The temperature derivative of heat flow of all non-eutectic samples shows temperature-dependent variations while that of the eutectic sample x =0.57 remains constant. The results mean that the phase transition takes place beyond the main peak region for the non-eutectic samples and it is therefore confirmed that the small particles in the x =0.57 nano-alloy sample, which contribute to the majority of the sample surface area, still remains eutectic after ultrasonication.

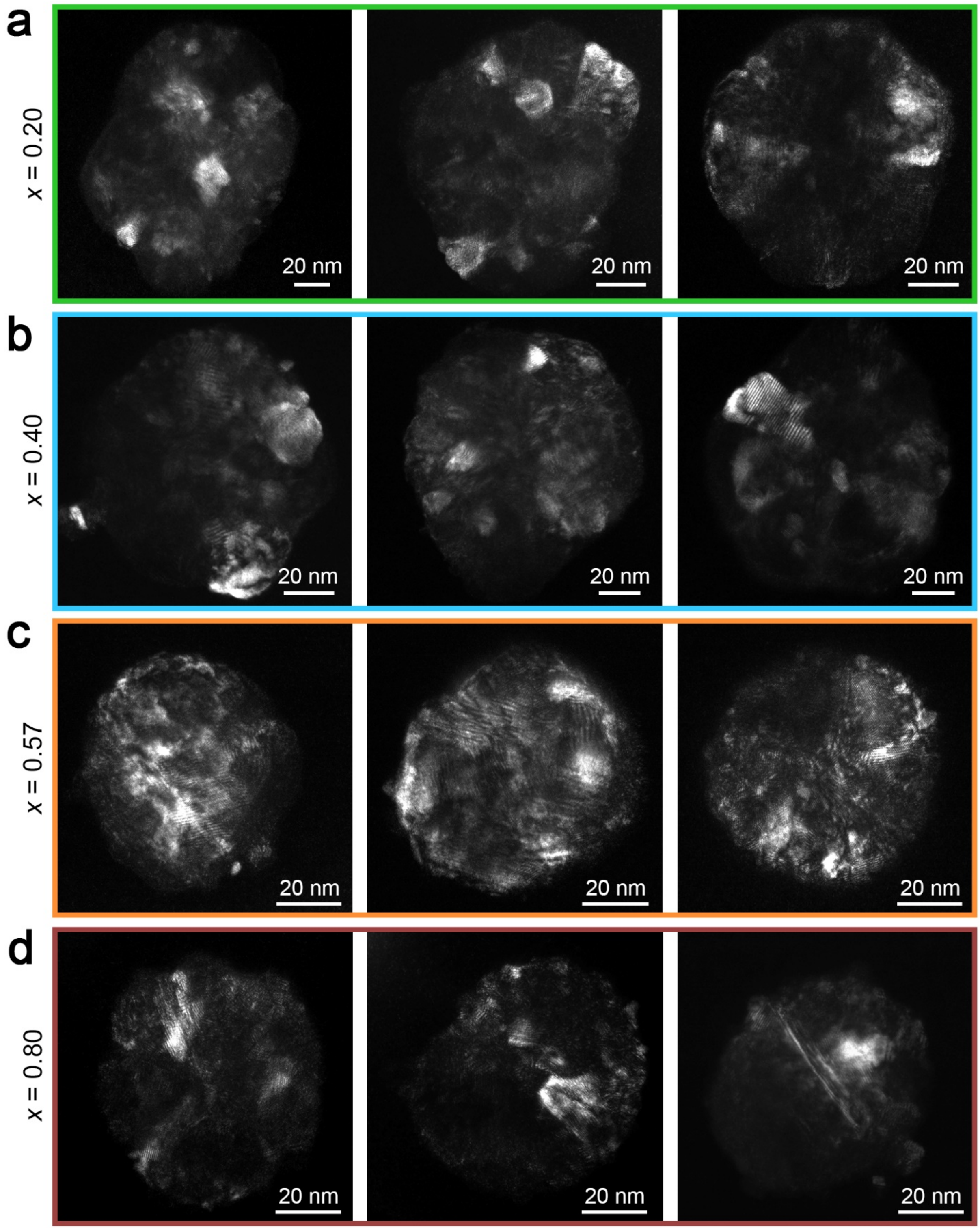

Supplementary Figure 9. Additional dark-field TEM images of different Bi_xSn_{1-x} nano-alloy samples with Bi ratio x indicated.

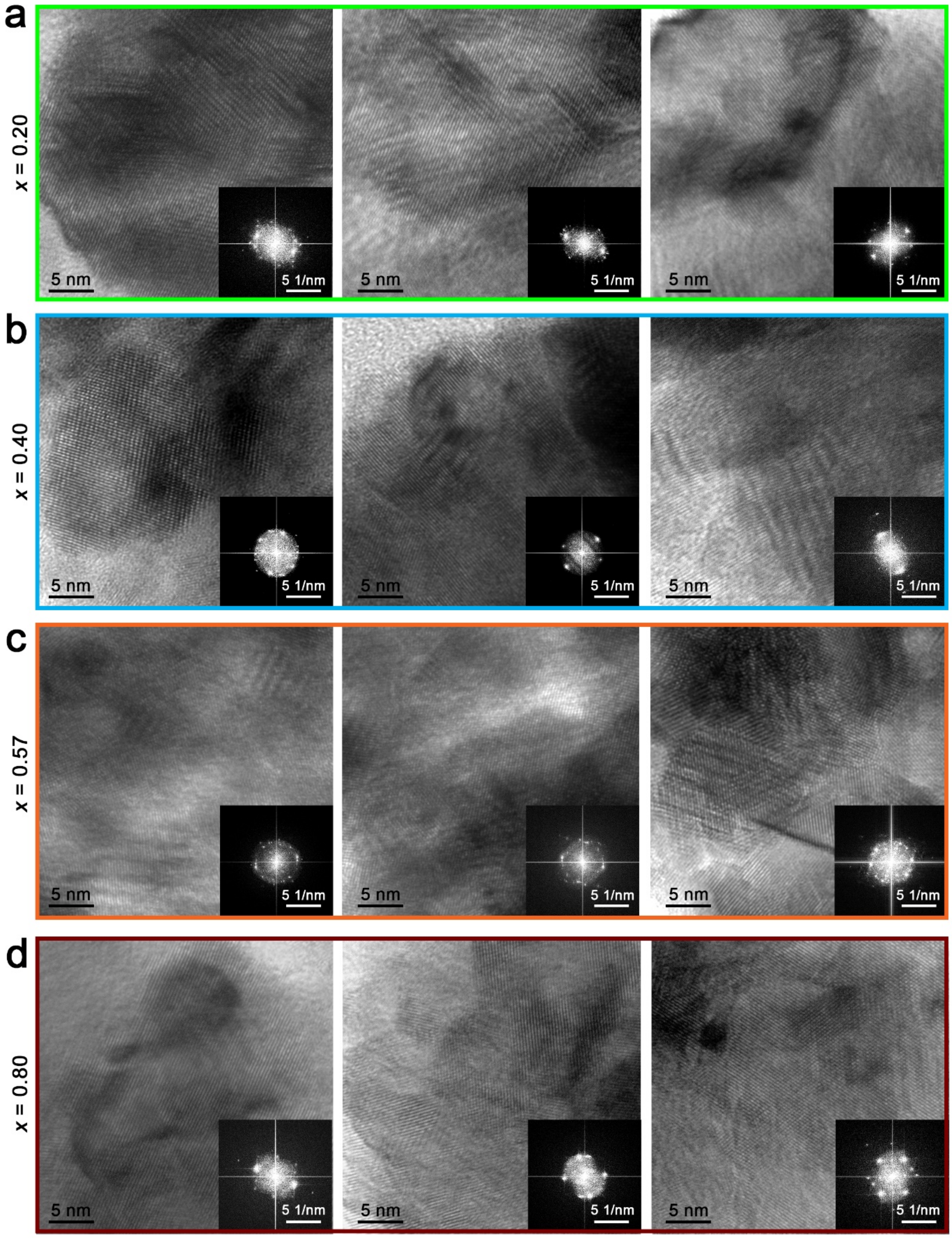

Supplementary Figure 10. Additional BF-HR-TEM images with the insets showing the FFTs of the Bi-Sn nano-alloys of different compositions as indicated. The eutectic nanoparticles typically show smaller grains than the non-eutectics and their omnidirectionally oriented grains render the FFT patterns circular.

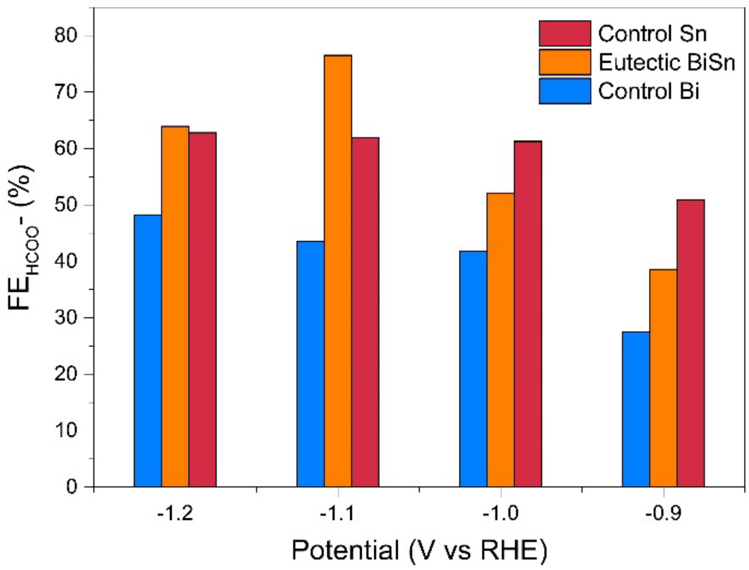

Supplementary Figure 11. Dependence of Faradaic efficiency for $HCOO^-$ on applied potential for eutectic Bi-Sn nano-alloy sample and control Sn and Bi samples.

Supplementary Table 1. CO_2RR catalytic performances of various Sn- and Bi-based catalysts.

Catalyst	Electrolyte	Operating potential (V vs RHE)	Current density (mA/cm^2)	Faradaic efficiency for $HCOO^-$ (%)	Reference
**Eutectic Bi-Sn**	**0.1 M $KHCO_3$**	**-1.1**	**10.7**	**78**	**This work**
m-SnO_2	0.1 M $KHCO_3$	-1.15	10.8	75.2	1
SnO_2/Graphene	0.1M $NaHCO_3$	-1.16	10.2	93.6	2
1D SnO_2 wire in tube	0.1 M $KHCO_3$	-0.99	7	63	3
Heat-treated Sn dendrite	0.1 M $KHCO_3$	–1.36	17.1	71.6	4
SnO_2 nanosheets on carbon cloth	0.5 M $NaHCO_3$	-0.99	45	87	5
Bi-Sn	0.5 M $KHCO_3$ with 600 rpm stirring	-1.14	-61	94	6
Sn/SnOx/Ti	0.5 M $NaHCO_3$	-0.7	1.8	55	6
Annealed Sn NPs	0.1M $KHCO_3$	-1.2	4	51.5	7
Bi nanosheets	0.5 M $NaHCO_3$	-0.8	-5	94	8
Sn quantum sheets/Graphene	0.1 M $NaHCO_3$	-1.15	21.5	89	9
SnO_2 nanowires (plasma treated)	0.1 M $KHCO_3$	-0.8	6	81	10

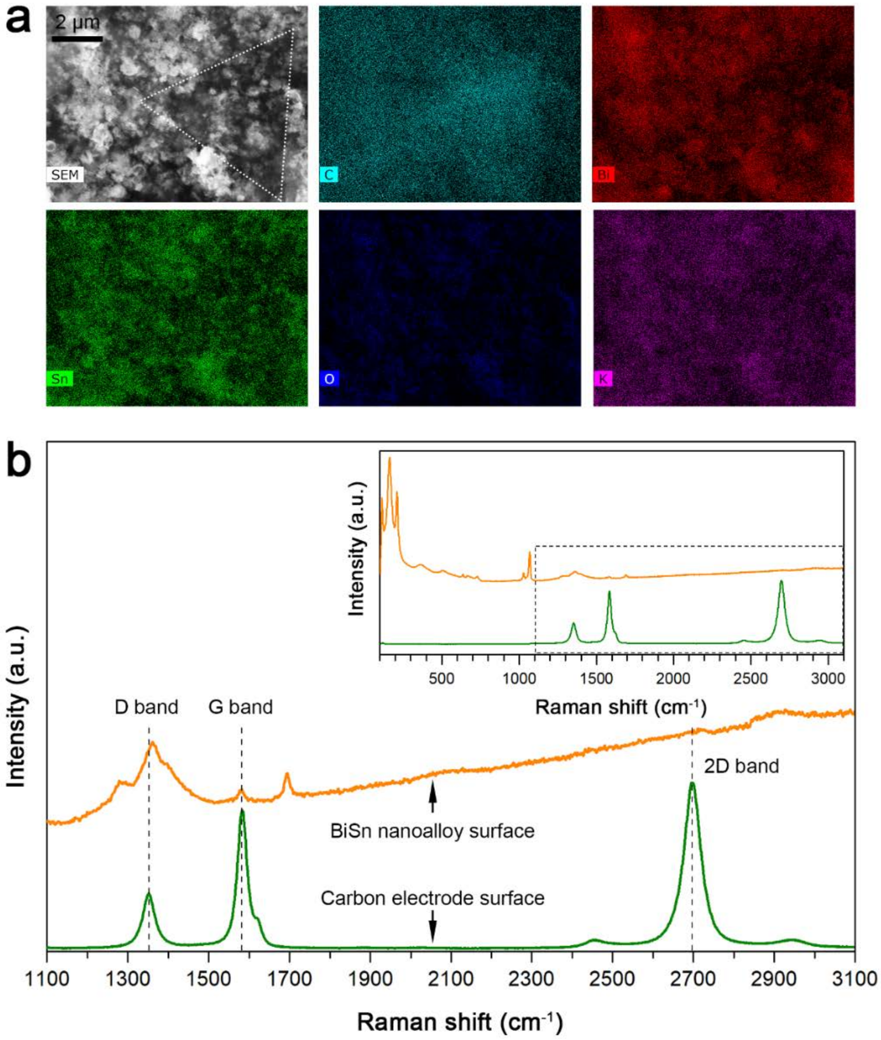

Supplementary Figure 12. Evidence for the formation of carbonaceous species during the CO_2RR reaction. **a** SEM-EDX mapping of the nano-alloy (eutectic) surface after CO_2RR reaction. The outlined triangle region, which is darker than the Bi-Sn nano-alloys under SEM, shows higher C density than other regions. This enrichment of C is supposed to be a result of direct reduction of CO_2 to solid C during the CO_2RR reaction. **b** Raman spectra collected from the Bi-Sn nano-alloy surface (yellow curve) and from the surface of the carbon electrode used to support the Bi-Sn nano-alloy (green curve). The inset shows the extended Raman spectra of **b**. When compared with the Raman spectra of the carbon electrode, the D band of the carbon material on the Bi-Sn nano-alloy sample is slightly shifted. In addition, it does not have a 2D band at near 2700 cm^{-1}. Such differences confirm that the carbon Raman signal should come from carbon species produced during the CO_2RR reaction, not from the carbon electrode.

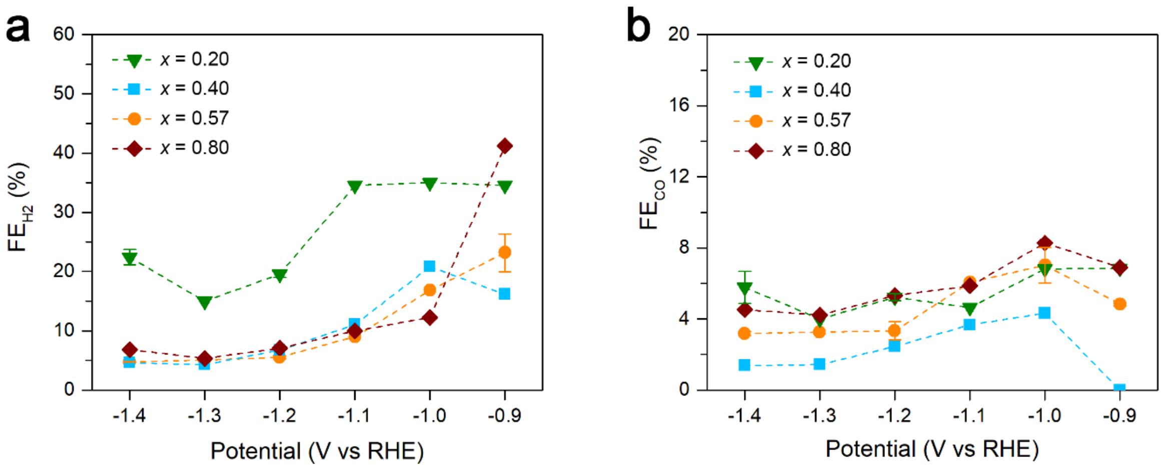

Supplementary Figure 13. a Dependence of the Faradic efficiency of H_2 and **b** that of CO on the applied potential for different Bi-Sn nano-alloy samples.

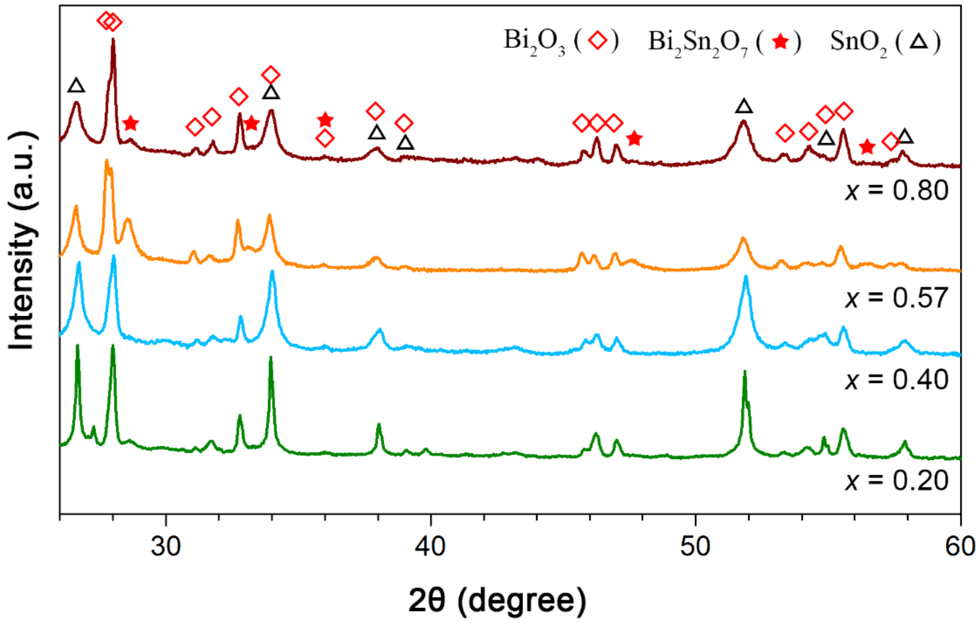

Supplementary Figure 14. XRD patterns of the annealed Bi-Sn nano-alloy samples with their starting Bi ratio indicated.

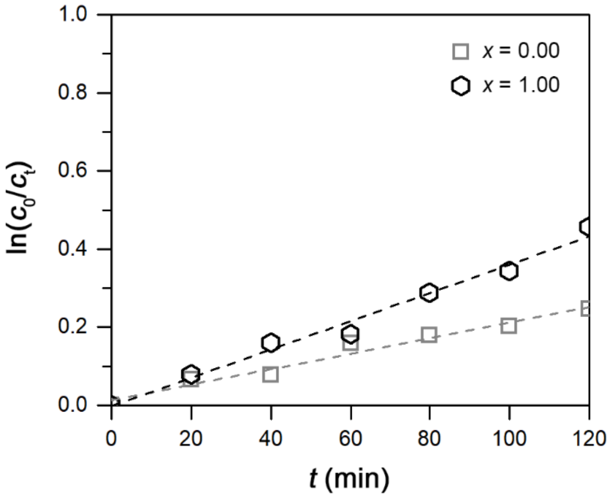

Supplementary Figure 15. Plots of ln (c_0/c_t) *vs* *t* for the control Sn (x = 0.00) and Bi (x = 1.00) samples.

Supplementary Table 2. Eutectic composition and melting point of some binary alloy systems[11].

Alloy system A-B	Eutectic composition $A_{wt\%}B_{wt\%}$	Melting point of A (°C)	Melting point of B (°C)	Eutectic melting point of A-B (°C)
Bi-Cd	$Bi_{60}Cd_{40}$	271.4	321.1	146
Bi-In	$Bi_{43.3}In5_{6.7}$	271.4	156.6	72.7
Bi-Li	$Bi_{23}Li_{77}$	271.4	180.6	175.0
Bi-Pb	$Bi_{55.2}Pb_{44.8}$	271.4	327.5	125.5
Bi-Pd	$Bi_{97}Pd_{3}$	271.4	1555	256
Bi-Pt	$Bi_{99.2}Pt_{0.8}$	271.4	1769	259
Bi-Sm	$Bi_{99}Sm_{1}$	271.4	1074	252
Bi-Sn	$Bi_{57}Sn_{43}$	271.4	232.0	139
Bi-Te	$Bi_{98.3}Te_{1.7}$	271.4	449.6	266
Bi-Yb	$Bi_{95}Yb_{5}$	271.4	819	250
Bi-Zn	$Bi_{97.3}Zn_{2.7}$	271.4	419.6	254.5
Cd-In	$Cd_{25.3}In_{74.4}$	321.1	156.6	126
Cd-Pb	$Cd_{17.5}Pb_{82.5}$	321.1	327.5	246
Cd-Sb	$Cd_{92}Sb_{8}$	321.1	630.8	290
Cd-Sn	$Cd_{32.25}Sn_{67.75}$	321.1	232.0	176
Cd-Tl	$Cd_{17}Tl_{83}$	321.1	304	203.5
Cd-Zn	$Cd_{82.6}Zn_{17.4}$	321.1	419.6	266
Cu-In	$Cu_{0.9}In_{99.1}$	1064.9	156.6	153
Cu-Tin	$Cu_{0.7}Sn_{99.3}$	1064.9	232.0	227
Dy-Sn	$Dy_{1.2}Sn_{98.8}$	1412	232.0	215
Ga-Ag	$Ga_{94.5}Ag_{5.5}$	29.8	951.9	25.0
Ga-In	$Ga_{78.6}In_{21.4}$	29.8	156.6	15.3
Ga-Mn	$Ga_{99}Mn_{1}$	29.8	1246	29.8
Ga-Sn	$Ga_{86.5}Sn_{13.5}$	29.8	232.0	20.5
Ga-Yb	$Ga_{99}Yb_{1}$	29.8	819	27
Ga-Zn	$Ga_{96.4}Zn_{3.4}$	29.8	419.6	24.7
Li-Pd	$Li_{51}Pd_{49}$	180.6	1555	145
Li-Sn	$Li_{98}Sn_{2}$	180.6	232.0	179
Li-Sr	$Li_{37}Sr_{63}$	180.6	769	134
Li-Tl	$Li_{77}Tl_{23}$	180.6	304	175
Li-Zn	$Li_{69.3}Zn_{30.7}$	180.6	419.6	161
Mg-Sn	$Mg_{2.1}Sn_{97.9}$	650	232.0	203.5
Mg-Tl	$Mg_{3}Tl_{97}$	650	304	202
Pb-Pd	$Pb_{95.5}Pd_{4.5}$	327.5	1555	260
Pb-Pt	$Pb_{95}Pt_{5}$	327.5	1769	290
Pb-Sb	$Pb_{88.9}Sb_{11.1}$	327.5	530.8	251.7
Pb-Sn	$Pb_{38.1}Sn_{61.9}$	327.5	232.0	183
Pd-Sn	$Pd_{99}Sn_{1}$	1555	232	230
Pd-Tl	$Pd_{99}Tl_{1}$	1555	302	293
Pt-Sn	$Pt_{0.8}Sn_{99.2}$	1769	232.0	226
Sb-Tl	$Sb_{19.7}Tl_{80.3}$	630.8	304	195
Se-Tl	$Se_{47.5}Tl_{52.5}$	221	304	199
Sn-Sr	$Sn_{99}Sr_{1}$	232.0	759	230
Sn-Tl	$Sn_{57}Tl_{43}$	232.0	304	166
Sn-Zn	$Sn_{91.2}Zn_{8.8}$	232.0	419.6	198.5
Te-Tl	$Te_{42}Tl_{58}$	449.6	304	224
Tl-Zn	$Tl_{97}Zn_{3}$	304	419.6	292

Supplementary Table 3. Properties of some proposed liquids for ultrasonication[12].

Liquid	Melting point	Boiling point	Solubility in water	Flammability
Water	0	100	-	Non-flammable
Dimethyl sulfoxide (DMSO)	19	189	Miscible	Flammable
Glycerol	17.8	290	Miscible	Flammable
Paraffin wax	37	> 370	Immiscible	Flammable
Silicone oil	< 0 (pour point)	> 300	Immiscible	Non-flammable
$AlBr_3$	97.5	257	Soluble	Non-flammable
$AlCl_3$-KCl (33:67 mole %)	90		Soluble	Non-flammable

Supplementary References

1 Daiyan, R., Lu, X., Saputera, W. H., Ng, Y. H. & Amal, R. Highly selective reduction of CO_2 to formate at low overpotentials achieved by a mesoporous tin oxide electrocatalyst. *ACS Sustain. Chem. Eng.* **6**, 1670-1679 (2018).

2 Zhang, S., Kang, P. & Meyer, T. J. Nanostructured tin catalysts for selective electrochemical reduction of carbon dioxide to formate. *J. Am. Chem. Soc.* **136**, 1734-1737 (2014).

3 Fan, L., Xia, Z., Xu, M., Lu, Y. & Li, Z. 1D SnO2 with wire-in-tube architectures for highly selective electrochemical reduction of CO_2 to C1 products. *Adv. Funct. Mater.* **28**, 1706289 (2018).

4 Won, D. H. *et al.* Rational design of a hierarchical tin dendrite electrode for efficient electrochemical reduction of CO_2. *ChemSusChem* **8**, 3092-3098 (2015).

5 Li, F., Chen, L., Knowles, G. P., MacFarlane, D. R. & Zhang, J. Hierarchical mesoporous SnO_2 nanosheets on carbon cloth: A robust and flexible electrocatalyst for CO_2 reduction with high efficiency and selectivity. *Angew. Chem. Int. Ed.* **56**, 505-509 (2017).

6 Chen, Y. & Kanan, M. W. Tin oxide dependence of the CO_2 reduction efficiency on tin electrodes and enhanced activity for tin/tin oxide thin-film catalysts. *J. Am. Chem. Soc.* **134**, 1986-1989 (2012).

7 Wu, J., Risalvato, F. G., Ma, S. & Zhou, X.-D. Electrochemical reduction of carbon dioxide III. The role of oxide layer thickness on the performance of Sn electrode in a full electrochemical cell. *J. Mater. Chem. A* **2**, 1647-1651 (2014).

8 Han, N. *et al.* Ultrathin bismuth nanosheets from in situ topotactic transformation for selective electrocatalytic CO_2 reduction to formate. *Nat. Commun.* **9**, 1320 (2018).

9 Lei, F. *et al.* Metallic tin quantum sheets confined in graphene toward high-efficiency carbon dioxide electroreduction. *Nat. Commun.* **7**, 12697 (2016).

10 Kumar, B. *et al.* Reduced SnO_2 porous nanowires with a high density of grain boundaries as catalysts for efficient electrochemical CO_2-into-HCOOH conversion. *Angew. Chem. Int. Ed.* **56**, 3645-3649 (2017).

11 Baker, H. & Okamoto, H. *ASM Handbook. Vol. 3. Alloy Phase Diagrams* (ASM International, 1992).

12 Lide, D. R. *CRC Handbook of Chemistry and Physics* Vol. 85(CRC Press, 2004).
